# Neuromuscular Electrical Stimulation during Hemodialysis Suppresses Postprandial Hyperglycemia in Patients with End-Stage Diabetic Kidney Disease: A Crossover Controlled Trial

**DOI:** 10.3390/jcm11216239

**Published:** 2022-10-22

**Authors:** Tomoki Tsurumi, Yuma Tamura, Yuki Nakatani, Tomoki Furuya, Hajime Tamiya, Masato Terashima, Takashi Tomoe, Asuka Ueno, Masahiro Shimoyama, Takanori Yasu

**Affiliations:** 1 Department of Rehabilitation, Dokkyo Medical University Nikko Medical Center, Nikko 321-2593, Japan; 2 Department of Diabetes and Endocrinology, Dokkyo Medical University Nikko Medical Center, Nikko 321-2593, Japan; 3 Department of Cardiovascular Medicine and Nephrology, Dokkyo Medical University Nikko Medical Center, Nikko 321-2593, Japan; 4 Social Participation and Community Health Research Team, Tokyo Metropolitan Institute of Gerontology, Itabashi, Tokyo 173-0015, Japan; 5 Department of Physical Therapy, Igaku Academy, Kawagoe 350-0003, Japan; 6 Institute for Human Movement and Medical Sciences, Niigata University of Health and Welfare, Niigata 950-3198, Japan

**Keywords:** diabetic kidney disease, neuromuscular electrical stimulation, glycemic control, hemodialysis

## Abstract

Hemodialysis patients with diabetic kidney disease (DKD) experience blood glucose fluctuations owing to insulin removal. We evaluated the effects of single and long-term application of neuromuscular electrical stimulation (NMES) during hemodialysis on glycemic control. This trial was conducted in two stages: Stage 1, following a crossover design and 4 week washout period, eleven outpatients with DKD either underwent a single bout of NMES for 30 min (NMES period) or rested (control period) after receiving nutritional support during hemodialysis; Stage 2, following a crossover design and 4 week washout period, each participant received the intervention for 12 weeks. NMES was administered for 30 min at the maximum tolerable intensity. The mean subcutaneous glucose concentration and mean amplitude of glycemic excursion (MAGE) were determined by flash glucose monitoring for 24 h. Changes in glycoalbumin and MAGE before and after NMES initiation were evaluated. The mean blood glucose level and MAGE after a single bout of NMES were significantly lower than those after rest. Glycoalbumin levels and echo intensity of the rectus femoris tended to decrease, but not significantly by ANOVA due to a lack in statistical power after the dropout of three patients. NMES in end-stage DKD decreased blood glucose levels during and after hemodialysis.

## 1. Introduction

The number of hemodialysis patients is increasing worldwide, with Type 2 diabetes mellitus accounting for the largest proportion of patients with primary renal disease [1]. Patients with diabetic kidney disease (DKD) undergoing hemodialysis develop insulin secretory defects along with insulin resistance, and their blood insulin levels may be reduced below normal [2]. Other significant changes in glycemic control occur during hemodialysis, such as the formation of a glucose gap between the blood and dialysate together with the diffusion of plasma glucose into the dialysate, resulting in a decrease in blood glucose levels [3]. A marked decrease in blood glucose levels during hemodialysis has been reported to induce hyperglycemia after the completion of hemodialysis [4]. Such rapid changes in blood glucose levels are associated with increased mortality as well as the risk of heart disease and require medical management [5].

Protein-energy wasting (PEW) is a state of catabolism due to metabolism and malnutrition in chronic disease [6], affecting about 28–54% of hemodialysis patients [7]. Hemodialysis is a method of removing metabolites and unnecessary substances, such as urinary toxins, from the body; however, it may inadvertently remove physiologically active substances, such as amino acids and pyruvic acid, which are necessary for skeletal muscle synthesis [8,9]. This may lead to sarcopenia, a complication of chronic kidney disease (CKD) [10]; skeletal muscle damage due to uremic toxin accumulation and PEW specific to patients with CKD is called uremic sarcopenia [11]. Among patients with CKD, 20% already have sarcopenia at the time of hemodialysis introduction. Muscle mass and muscle strength decrease markedly as the hemodialysis period increases [12].

To maintain or improve muscle function, neuromuscular electrical stimulation (NMES) has been used as an alternative to exercise therapy. NMES has been reported to lower blood glucose levels by predominantly stimulating type II fibers in diabetic patients [13], to improve glucose tolerance [14], and to promote muscle protein synthesis in elderly people with diabetes [15]. A systematic review regarding long-term NMES application [16] showed that NMES increased knee extension strength, 6 min walking distance, chair standing test score, and grip strength in hemodialysis patients in their 40s to 70s. Moreover, oral amino acid supplementation can improve serum levels of albumin and total protein in hemodialysis patients with hypoalbuminemia [17]. These results suggest that the supplementation of amino acids—which are necessary for skeletal muscle synthesis but are leaked during hemodialysis—and skeletal muscle exercise using NMES may be effective in improving sarcopenia in patients with DKD undergoing hemodialysis. However, amino acid supplementation during hemodialysis is not routinely recommended, and its effects when used in combination with NMES have not yet been elucidated.

To fill this gap in the literature, we looked at the effect of a single bout of NMES after nutritional supplementation with amino acids on blood glucose fluctuations in patients with DKD on hemodialysis. We also investigated the improvement of skeletal muscle function and stabilization of glycemic control after long-term (12 week) intervention with NMES.

## 2. Materials and Methods

### 2.1. Participants

This prospective crossover study included DKD patients on hemodialysis who attended Dokkyo Medical University Nikko Medical Center between July 2017 and December 2020. Patients who were between 50 and 90 years of age at the time of consent, required hemodialysis three times/week, and who were prescribed a hypoglycemic agent and/or insulin for diabetes were eligible for participation. In addition, the type of drug and insulin dose were not changed during the study period. Of the 32 eligible patients, 11 provided written informed consent. The clinical characteristics of these 11 patients are presented in Table 1. The study excluded patients with contraindications to NMES use such as pacemaker insertion, patients with contraindications to exercise therapy—as described in the American College of Sports Medicine’s Guidelines for Exercise Testing and Prescription, 9th edition [18]—patients with acute illness or cancer, limb deficiencies, difficulty in walking independently, as well as those with skin diseases, wounds, and pruritus, which made it difficult to perform NMES. The Ethics Committee of Dokkyo Medical University Nikko Medical Center approved the research protocol (Approval number: Nikko 29005). This study was registered in the University Hospital Medical Information Network Clinical Trials Registry (Identifier UMIN000039310).

### 2.2. Study Design

The study was conducted in two stages. Both stages (1 and 2) were conducted as single-center, crossover, and controlled trials (Figure S2). Cross-over allocation was performed by generating a random number table in Excel and randomly determining the order of interventions using the substitution block method.

### 2.3. Sample Size

The sample size calculation was performed using G*Power (Heinrich-Heine-University Düsseldorf, North Rhine-Westphalia, Germany). The number of study participants was set based on a study in which NMES intervention was performed in patients with diabetes mellitus 30 min after a meal [13]. The target number of patients was 11 (α = 0.05, 1 − β = 0.95).

### 2.4. Neuromuscular Electrical Stimulation Procedure

Five rubber belt electrodes were attached to each of the lower limbs and abdomen (lumbar, 5.3 × 93.3 cm; thigh, 5.3 × 69.6 cm; ankle, 5.3 × 54.6 cm; Homer ion, Tokyo, Japan). All muscles were simultaneously contracted for 30 min by an exponential growth wave of 250 ms pulses at a frequency of 4 Hz. The stimulus intensity was individually set at the maximum intensity that each participant could tolerate. Pulse rate and blood pressure before and after NMES were monitored during all sessions.

### 2.5. Blood Sample and Flash Glucose Monitoring Analysis

Blood samples were collected from the bleeding side of the arteriovenous shunt before heparin infusion. Blood glucose levels were measured from whole blood samples using FreeStyle Libre (Abbott, IL, USA). Blood lactate concentration was measured using the lactate oxidase method with an automated analyzer (Lactate Pro; Arklay, Kyoto, Japan). Blood samples were collected in tubes, and glycoalbumin, high-density lipoprotein cholesterol, low-density lipoprotein cholesterol, and albumin were measured (BML, Inc., Tokyo, Japan). The Geriatric Nutritional Risk Index (GNRI) was calculated as GNRI = 14.89 × serum albumin (g/dL) + 41.7 × (current weight/ideal weight) ideal weight (kg) = height (m^2^) × 22 [19]. Subcutaneous glucose concentrations levels were assessed by self-measurement using FreeStyle Libre (Abbott, IL, USA). The mean subcutaneous glucose level and mean amplitude of glycemic excursion (MAGE) were analyzed using the glucose concentration in the subcutaneous interstitial fluid every 15 min by Flash Glucose Monitoring (FGM). MAGE was the subcutaneous glucose concentration obtained from FGM at 24 h after the end of dialysis. MAGE was calculated using EasyGV version 9.0 (EasyGV by Nathan R Hill. © University of Oxford 2010+) [20]. The plasma levels of derivatives of reactive oxygen metabolites (d-ROMs) and the biological antioxidant potential (BAP) were assessed using d-ROM and BAP test kits (Diacron International, Grosseto, Italy). Evaluators were blinded to patient data and period.

### 2.6. Muscular and Physical Functions

Muscle function was assessed quantitatively by measuring thickness, which indicated skeletal muscle mass, and qualitatively by echo intensity. The participants were placed in a relaxed supine position, and an ultrasound device (XARIO XG SSA-680A; TOSHIBA Medical Systems, Tokyo, Japan) was used to measure the linear-array probe. The ultrasound device was set to the B mode with a frequency of 12 Hz, 60-dB dynamic range, 85-dB gain, and 6 cm depth; the measurements were taken by a trained examiner. The thickness of the rectus femoris and vastus intermedius muscles on the anterior surface of the thigh was recorded. Echo intensity was measured on the rectus femoris and evaluated using Image-J with an 8-bit grayscale. The skeletal muscle mass index (SMI) was measured using a bioelectrical impedance data acquisition system (MC-180; TANITA, Tokyo, Japan) after completion of hemodialysis. Maximal isometric knee extension strength was measured twice using a hand-held dynamometer (Mobie MT-150; MINATO, Tokyo, Japan) at 90° knee joint flexion, and the higher value was adopted. Grip strength was measured twice with the dominant hand using a grip strength meter (Drip-D; Takei, Niigata, Japan), and the higher value was used to calculate the weight-bearing index. The 6 min walking test (6MWT) measured the total walking distance over a period of 6 min. In the timed up and go test, the patients were timed while they rose from a chair, walked 3 m away, turned and walked back to the chair, then sat down again. The Short Physical Performance Battery (SPPB) measured side-by-side, semi-tandem, and tandem positions, the time taken to walk 4 m, as well as time taken to rise from a chair and return to a seated position five times. In accordance with the sarcopenia diagnostic criteria reported by Chen et al. in 2019 [21], muscle strength was defined as grip strength < 28 kg for men and <18 kg for women, physical performance criteria were an SPPB score ≤ 9 or five-time chair stand test duration ≤12 s, whereas muscle mass was defined as <7.0 kg/m^2^ for men and <5.7 kg/m^2^ for women in the bioelectrical impedance analysis. Low muscle strength and muscle mass or low physical function with low muscle mass was defined as sarcopenia. Evaluators were blinded to the patient data and period allocation process.

### 2.7. Experimental Protocol

#### 2.7.1. Stage 1

This stage of the trial examined the effect of a single bout of NMES during hemodialysis on glycemic control. Following a crossover design and 1 week washout period, the 11 participants either received NMES for 30 min (NMES period) or rested (control period) after receiving nutritional support during hemodialysis (Figure S1). Participants were instructed to lead a normal life during the study period, as dietary changes and exercise would affect their glycemic control if they became habitual. Participants were acclimatized as they underwent NMES three times during hemodialysis 2 weeks before the start of the study. An FGM sensor was attached to the posterior surface of the upper arm 3 days before the test day. Participants ate breakfast and took their oral medication as they would during a normal hemodialysis session before the test. Blood samples were collected before the start of hemodialysis after puncturing the indwelling needle, and nutritional support was provided 30 min after the start of hemodialysis. Nutritional support was provided orally for both the NMES and control periods. Two nutritional supplements (300 kcal [protein, 15.0 g; total fat, 0 g; carbohydrate, 61.5 g; sodium, 104 mg; potassium, 12 mg; phosphorous, 74 g; Rehatime Jelly, CLINICO, Tokyo, Japan; Enjoy Argina, CLINICO, Tokyo, Japan]) were provided. Blood samples were repeated at 30, 60, 90, 120, and 150 min after the start of hemodialysis and at completion. In the NMES period, NMES was performed for 30 min starting at 60 min after the start of hemodialysis. In the control period, blood was collected using the same protocol, and patients were placed in bed at rest for 30 min to 60 min after the start of hemodialysis. Blood pressure and pulse rate were measured every 30 min during the study, and body weight along with skeletal muscle mass were measured after the completion of hemodialysis. The room temperature was maintained at 24–26 °C.

#### 2.7.2. Stage 2

After stage 1 was completed, stage 2 of the trial was conducted to examine the long-term (12-week) effect of NMES during hemodialysis on blood glucose control. A crossover design was followed with a 4 week washout period, and participants either underwent NMES (NMES period) or rested (control period) after receiving nutritional support during hemodialysis (Figure S1). The intervention (NMES) was performed three times a week for 30 min during hemodialysis for 12 weeks. Supplemental nutrition, such as an amino acid supplements, was not administered orally during hemodialysis because it could have affected glycemic control and thus confounded the results of this trial. The participants were instructed to lead a normal life during the study period, as dietary changes and exercise would affect their glycemic control if they became habitual.

### 2.8. Statistical Analyses

Statistical analysis of the single bout of NMES was performed using two-way repeated measures analysis of variance (ANOVA) to examine changes in blood glucose, lactic acid, C-peptide, systolic blood pressure, and pulse rate over time along with differences between the two study periods (control vs. NMES). ANOVA was used to determine whether a trial–time interaction was found. One-way ANOVA was used for each parameter to evaluate the difference between trajectories at each time point. All post-hoc tests were performed using the Tukey method, when *p* < 0.05 was detected in ANOVA. The FGM was examined using paired *t*-test. For the 12-week effect analysis, various parameters were compared using a repeated two-way ANOVA. The amount of change was calculated by subtracting the initial values from the values at 12 weeks, which was compared using a paired *t*-test. All statistical analyses were performed using the JMP Pro 14 (SAS Institute Inc., Cary, NC, USA), with significance set at *p* < 0.05.

## 3. Results

### Effect of a Single Bout of NMES

**Figure 1:**
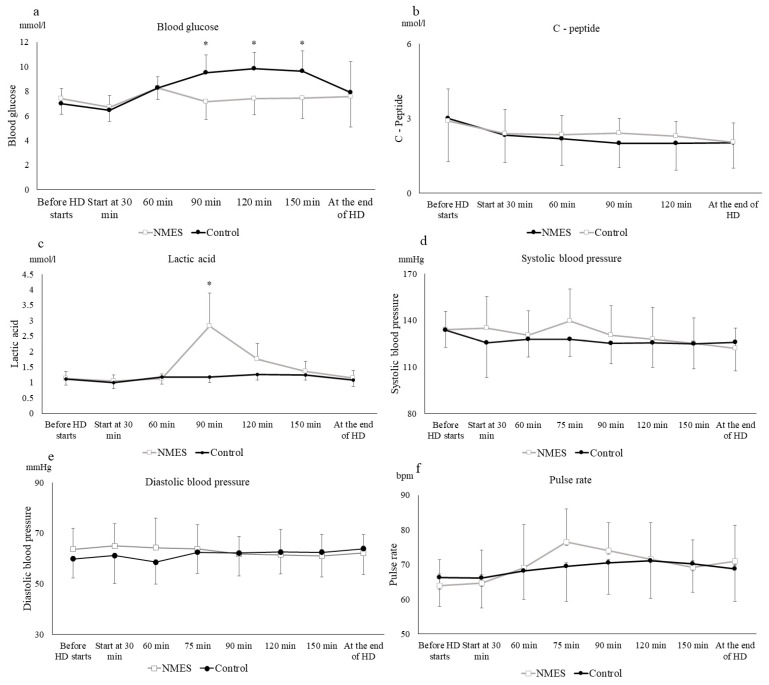
Serial change of each parameter in stage 1. Serial changes in blood levels of glucose (**a**), C-peptide (**b**), lactic acid (**c**), along with systolic blood pressure (**d**), diastolic blood pressure (**e**), and pulse rate (**f**) by a single bout of neuromuscular electrical stimulation (NMES) implemented after supplementary nutrient inoculation in 11 hemodialysis patients studied on two occasions: black circles, control trial; white squares, neuromuscular electrical stimulation (NMES) trial. All the participants took nutritional supplements 30 min after the start of hemodialysis (HD). NMES was performed from 60 min after the start of hemodialysis until 90 min. Values represent the mean ± standard deviation for the 11 participants. * *p* < 0.01 vs. control.

**Figure 2:**
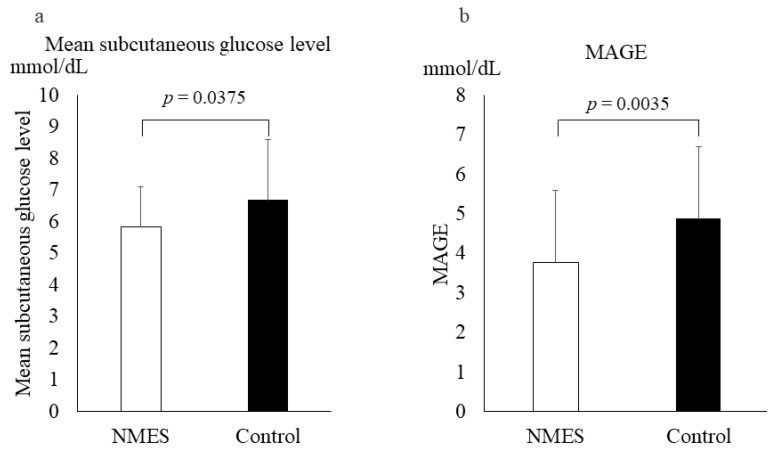
(**a**) Mean subcutaneous glucose level and (**b**) mean amplitude of glycemic excursion (MAGE) for 24 h after completion of hemodialysis with a single bout neuromuscular electrical stimulation (NMES) session after supplementary nutritional food intake in stage 1; NMES: white bars, control: black bars. Values represent the mean ± standard deviation of the 11 participants. Mean blood glucose level after hemodialysis for >24 h. Paired *t*-test of Δvalues when comparing differences between periods. Effect of the 12 week NMES on Glycemic Control.

There was a significant interaction between the blood glucose levels and lactic acid in each period and the blood collection time points. The blood glucose level in the NMES period was significantly lower than that in the control period at 90 min (7.1 ± 0.9 mmol in the NMES period, 9.5 ± 1.3 mmol in the control period), and 120 min (7.1 ± 0.9 mmol in the NMES period, 9.5 ± 1.3 mmol in the control period), *p* < 0.01 (Figure 1a). Lactic acid was transiently increased just after NMES at 90 min of hemodialysis (2.8 ± 1.1 mmol) in the test period but remained unchanged in the control period (1.2 ± 0.2 mmol), *p* < 0.01 (Figure 1b). There were no significant differences in C-peptide levels, systolic blood pressure, diastolic blood pressure, and pulse rate between the NMES and control periods (Figure 1c–f). Figure 1 and Figure 2 compare NMES-treated patients with untreated patients in Stage 1 of the study.

The mean subcutaneous glucose level at 24 h post-hemodialysis was significantly lower in the NMES period (5.8 ± 1.3 mmol) than in the control period (6.7 ± 1.9 mmol, *p* = 0.0375) as shown in Figure 2a. Additionally, MAGE was significantly lower in the NMES period (3.8 ± 1.8 mmol) than in the control period (4.8 ± 1.8 mmol, *p* = 0.0035) at 24 h post-hemodialysis (Figure 2b).

There were not significant differences in any of the measures during the NMES and control periods by ANOVA. However, glycalbumin levels and echo intensity of the rectus femoris tended to decrease (Table 2). Comparing the change between measurements, there was a greater reduction in glycoalbumin levels in the NMES period than in the control period (NMES period, −1.0 ± 0.9%; control period, −0.1 ± 0.9%; *p* = 0.0474). An increase in echo intensity of the rectus femoris was observed in the control period; however, a decrease was observed in the NMES period. These changes were statistically significant (NMES period, −12 ± 11.3; control period, 1.6 ± 5.4, *p* = 0.0207).

## 4. Discussion

To the best of our knowledge, this is the first study to demonstrate short- and long-term effects of NMES on glucose metabolism during hemodialysis in patients with end-stage DKD. In a single bout, NMES suppressed the increase in blood glucose after nutritional support during hemodialysis and reduced MAGE at 24 h after completion of hemodialysis compared with that in the control period, despite no change in serum C-peptide levels, which were used as an indicator of endogenous insulin secretion. In addition, the long-term (12 week) application of the intervention (NMES during hemodialysis) improved the glycoalbumin levels and echo intensity of the rectus femoris, although muscle strength and muscle mass remained unchanged.

Miyamoto et al., have already reported the insulin-independent suppression of blood glucose elevation by a single bout of NMES in patients with Type 2 diabetes mellitus without DKD [13]. The present study showed similar suppression of blood glucose elevation by NMES during hemodialysis in end-stage DKD patients. In addition, we found that during hemodialysis, NMES reduced MAGE and mean subcutaneous glucose concentrations after hemodialysis completion, suggesting that it remains effective for approximately 24 h after its administration. In healthy strength-trained men, voluntary exercise has also been reported to reduce fasting blood glucose level at 24 h after intervention [22], and we believe that the same effect occurs with NMES. In addition, patients with a MAGE of 3.9 mmol/L or higher have a higher incidence of cardiac events than those with less than 3.9 mmol/L [5]. The MAGE in the present study was below the cutoff value before as well as after the single bout of NMES, suggesting a clinically meaningful acute effect that may contribute to the reduction of cardiac events. GLUT4 translocation by NMES-induced muscle contraction may explain the main mechanism for the suppression of blood glucose elevation by NMES [23]. Lauritzen et al. have reported that in living mice, NMES-induced muscle contractions increase GLUT4 translocation to the sarcolemma and t-tubules with similar kinetics, and do not require AMPKα2 activity [24]. NMES-induced muscle contraction differs from voluntary exercise in that type II fiber motor units are preferentially contracted during NMES [25]. In other words, type II fibers with low mitochondrial content contract more predominantly during NMES. Thus, more lactic acid is released during muscle contraction by NMES. In the present study, a steep increase in lactic acid levels was observed immediately following NMES.

Long-term (12-week) administration of the intervention (NMES during hemodialysis) tended to improve glycoalbumin levels from 18.6 ± 1.7% to 17.4 ± 1.4%, but this not significant because of a lack in statistical power after the dropout of three patients. An 8 week crossover study with NMES intervention among diabetic patients without DKD reported no significant improvement in hemoglobin A1c (HbA1c) [26]. Our data in end-stage DKD on maintenance hemodialysis are in line with their results. However, a 4 week NMES study in obese healthy sedentary Hispanic subjects reported significant improvements of glucose tolerance [14]. In Stage 2, MAGE was taken 72 h after NMES, because of which there is a possibility that there was no difference in MAGE. Regarding the long-term effects, from the results of Stage 1, continuation of NMES lowered blood glucose levels and may lower GA.

NMES tended to improve the echo intensity of rectus femoris levels from 84.1 ± 11.3 to 71.2 ± 11.9, but muscle function and physical function parameters, such as knee extension strength, did not improve significantly. Muscle echo intensity is considered a qualitative assessment of skeletal muscle and is related to the infiltration of non-contractile tissues such as fat and fibrous tissue [27]. There were three dropouts in our study, so it is possible that ultrasound echo intensity in the rectus femoris was not statistically significant. It is possible that muscle strength will improve with further continuation of NMES. In fact, a 20 week, twice-weekly intervention study using NMES in hemodialysis patients reported improved knee extensor strength and prolonged 6MWT [28,29].

This study has some limitations. First, the sample size was limited to a single-center analysis. Second, participants were not served the same diet outside of hemodialysis during the study period. Although we instructed the participants to continue life as usual, we did not assess their daily physical activities.

## 5. Conclusions

NMES in patients with end-stage DKD decreased blood glucose level during and after hemodialysis. There is a possibility that continuing NMES application during hemodialysis improves glycoalbumin and echo intensity in the rectus femoris.

## Figures and Tables

**Table 1:** Physical characteristics, duration of hemodialysis, and metabolic parameters of participants.

Parameter	*n* = 11
Age (years)	74.0 ± 5.2
Male/female (*n*)	7/4
Duration of HD (months)	32.9 ± 20.0
BMI (kg/m²)	22.7 ± 3.9
SMI (kg/m²)	6.9 ± 0.9/5.5 ± 0.3
Glycoalbumin (%)	18.7 ± 1.7
HDL-cholesterol (mg/dL)	10.2 ± 2.0
LDL-cholesterol (mg/dL)	15.4 ± 4.9
Triglyceride (mg/dL)	13.2 ± 4.8
GNRI	92.0 ± 4.2
Sarcopenia (*n*)	10
Medication	
Insulin injection (*n*)	3
GLP-1 receptor agonist (*n*)	2
DPP-4 inhibitor (*n*)	4
α-GI (*n*)	4

Values represent the mean ± standard deviation of the 11 participants. Abbreviations: α-GI, α-glucosidase inhibitor; BMI, body mass index; DPP-4 inhibitor, dipeptidyl peptidase IV inhibitor; GLP-1 receptor agonist, glucagon-like peptide receptor agonist; GNRI, Geriatric Nutritional Risk Index; HD, hemodialysis; HDL, high-density lipoprotein; LDL, low-density lipoprotein; SMI, skeletal muscle index.

**Table 2:** Clinical variables recorded in stage 2 of the trial.

	Control		NMES		Repeated Measures 2-Way ANOVA			
	Pre	Post	Pre	Post	Group	Time	Interaction	
							F Value	*p* Value
Body composition								
Body weight (kg)	58.5 ± 15.4	58.4 ± 15.4	58.5 ± 15.3	59.0 ± 14.9	0.9726	0.9507	0.0028	0.9543
BMI (kg/m²)	23.8 ± 4.5	23.8 ± 4.6	23.9 ± 4.5	24.1 ± 4.4	0.9481	0.916	0.0073	0.9373
SMI (kg/m²)	6.5 ± 1.0	6.5 ± 0.9	6.4 ± 1.0	6.4 ± 1.0	0.8368	0.8449	0.0327	0.9005
Glycemic control								
Glycoalbumin (%)	18.6 ± 1.3	18.4 ± 1.8	18.6 ± 1.7	17.4 ± 1.4	0.1297	0.1297	1.9478	0.1568
Lipids								
HDL-cholesterol (mmol/L)	1.06 ± 0.26	1.15 ± 0.27	1.06 ± 0.20	1.13 ± 0.30	0.3778	0.8755	0.2844	0.8755
LDL-cholesterol (mmol/L)	1.52 ± 0.48	1.45 ± 0.41	1.54 ± 0.41	1.50 ± 0.42	0.7120	0.7596	0.0940	0.8246
Triacylglcerol (mmol/L)	1.30 ± 0.51	1.12 ± 0.47	1.26 ± 0.33	1.10 ± 0.42	0.9279	0.2826	0.4118	0.8707
Nutritional status indicators								
GNRI	93.1 ± 4.4	94.6 ± 2.9	93.3 ± 2.9	94.0 ± 3.6	0.3984	0.3871	0.5318	0.7267
FGM glucose over 24 h								
Mean (mmol/dL)	6.9 ± 1.6	7.0 ± 1.8	6.0 ± 1.3	6.4 ± 1.2	0.2447	0.5692	0.6961	0.5692
MAGE (mmol/dL)	4.0 ± 1.1	5.0 ± 2.2	4.2 ± 1.0	4.6 ± 1.8	0.2225	0.8747	0.6044	0.6437
Oxidative stress								
d-ROMs (U. CARR)	354.2 ± 24.4	350.3 ± 17.9	342.7 ± 24.5	326.8 ± 17.9	0.2040	0.288	2.5423	0.4361
BAP (μmmol/L)	2647.2 ± 313.2	2818.2 ± 200.7	2787.6 ± 376.5	2761.2 ± 234.9	0.4858	0.6870	0.5313	0.3433
Muscle function								
Echo intensity in the rectus femoris	78.8 ± 11.3	80.4 ± 10.7	84.1 ± 11.3	71.2 ± 11.9	0.1732	0.6335	1.8301	0.0798
Quadriceps muscle thickness (cm)	2.1 ± 0.4	2.0 ± 0.3	2.1 ± 0.5	2.1 ± 0.6	0.8884	0.9055	0.0617	0.7007
Grip power (kg)	20.5 ± 4.8	19.8 ± 5.8	19.7 ± 4.8	21.1 ± 6.2	0.9093	0.8426	0.1157	0.5921
Weight bearing index	0.33 ± 0.11	0.31 ± 0.0.8	0.30 ± 0.10	0.34 ± 0.10	0.9430	0.5956	0.3385	0.4026
Physical function								
SPPB (point)	9.1 ± 3.3	9.0 ± 3.5	8.6 ± 3.5	8.8 ± 3.5	0.8015	0.9599	0.0301	0.8801
TUG (s)	16.0 ± 18.9	17.0 ± 18.7	16.7 ± 18.0	13.3 ± 10.1	0.8060	0.8404	0.0792	0.7164
6WMT (m)	257.7 ± 148	257.9 ± 145.9	261.9 ± 148	277.4 ± 150.0	0.8225	0.8824	0.0316	0.8851
Sarcopenia (n)	6 (75%)	7 (88%)	7 (88%)	6 (75%)				

Data are presented as mean ± standard deviation. Abbreviations: 6MWT, 6 min walking test; BAP, biological antioxidant potential; BMI, body mass index; d-ROMS, diacron-reactive oxygen metabolites; FGM, flash glucose monitoring; GNRI, Geriatric Nutritional Risk Index; HDL, high-density lipoprotein; LDL, low-density lipoprotein; MAGE, mean amplitude of glycemic excursion; NMES, neuromuscular electrical stimulation; SMI, skeletal muscle index; SPPB, Short Physical Performance Battery; TUG, timed up and go.

## Data Availability

Not applicable.

## Supplementary Materials

The following information can be downloaded at https://www.mdpi.com/article/10.3390/jcm11216239/s1, Figure S1: A flow chart showing the participants throughout the trial. Abbreviations: NMES, neuromuscular electrical stimulation; Figure S2: Experimental protocol for single effect. The 11 participants either received NMES for 30 min (NMES period) or rested (control period) after receiving nutritional support during hemodialysis. Blood samples were collected before the start of hemodialysis after puncturing the indwelling needle, and nutritional support was provided 30 min after the start of hemodialysis. Blood pressure and pulse rate were measured every 30 min during the study. Nutritional support was provided orally for both NMES and control periods after the start of hemodialysis. FGM was used for 24 h after the end of dialysis as the scope of the analysis. Abbreviations: NMES, neuromuscular electrical stimulation; FGM, Flush glucose monitoring.
