# Supplementary material for: Neuromuscular Electrical Stimulation during Hemodialysis Suppresses Postprandial Hyperglycemia in Patients with End-Stage Diabetic Kidney Disease: A Crossover Controlled Trial
Source: J Clin Med. 2022 Oct 22;11(21):6239. doi: 10.3390/jcm11216239 (PMC9658571; doi:10.3390/jcm11216239)
Supplement: Supplementary file 1 [file jcm-11-06239-s001.zip › jcm-1913293-supplementary.pdf]

SUPPLEMENTARY MATERIAL

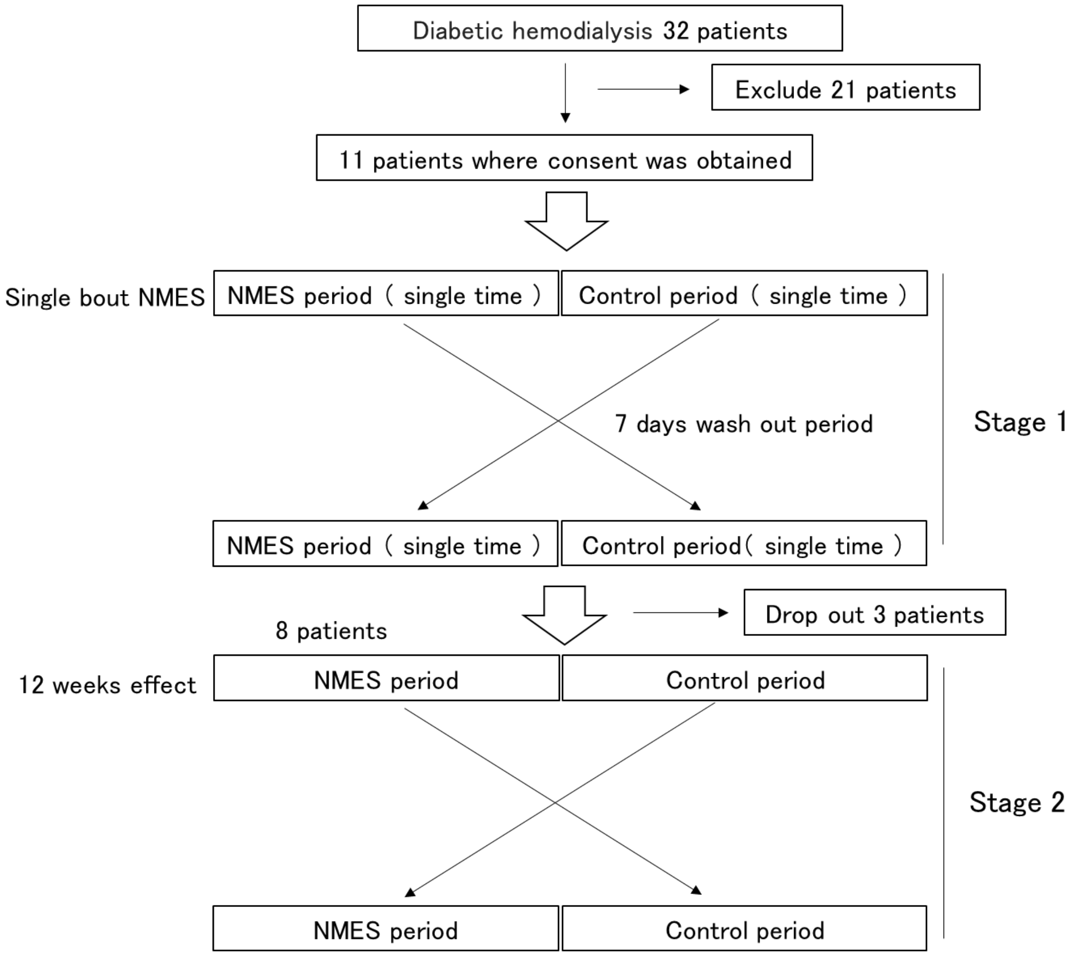

Figure S1. A flow chart showing the participants throughout the trial. Abbreviations: NMES, neuromuscular electrical stimulation.

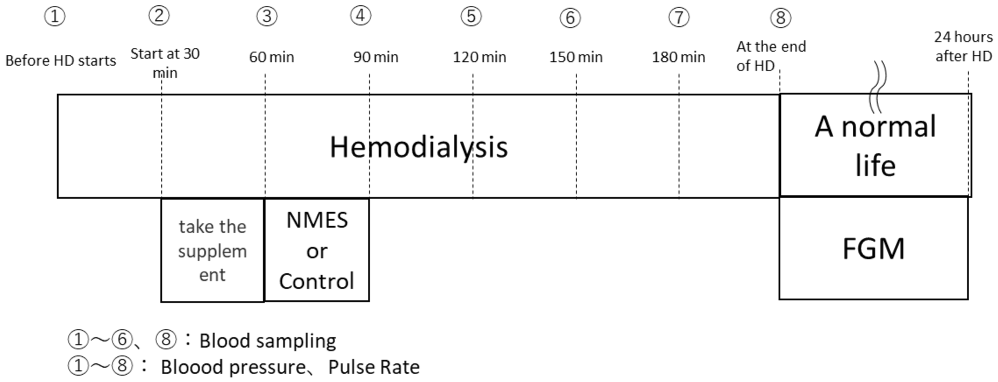

Figure S2. Experimental protocol for single effect. The 11 participants either received NMES for 30 min (NMES period) or rested (control period) after receiving nutritional support during hemodialysis. Blood samples were collected before the start of hemodialysis after puncturing the indwelling needle, and nutritional support was provided 30 min after the start of hemodialysis. Blood pressure and pulse rate were measured every 30 min during the study. Nutritional support was provided orally for both NMES and control periods after the start of hemodialysis. FGM was used for 24 h after the end of dialysis as the scope of the analysis. Abbreviations: NMES, neuromuscular electrical stimulation; FGM, flush glucose monitoring.
